# When Philosophers Differ

**Published:** 1871-08

**Authors:** 


### Editorial.

### WHEN PHILOSOPHERS DIFFER?

"The Statical motion holds dominion in the Mineral kingdom; the Vibratile, in the Vegetable, and the Locomotive in the Animal, and are correlated to Crystallosophy, Cellosophy, and Corpusculosophy, as the differentiated expressions of organic planetary forces."—*Dental Cosmos*, p. 372, vol. XIII.

That is a clear expression of the doctrine we have always advocated; and we have been sadly discouraged to find the profession so slow to recognize it. Only a few bright intellects (like the speaker quoted,) seem to have profited by our teachings; and, now, Blinks, who has gone crazy over the physiology and pathology of steam engines, and who, consequently, claims to speak only as the spirits of James Watt, Fulton, Black, Bolton, and others, give him utterance, tells us that "statical motion," (which he says is the motion bodies have when perfectly still,) is best illustrated by a waiting train on a "side track," or a steamboat on the docks for repairs; the vibratile, or "to and fro," he says, is perfectly illustrated by the yard engine, and the ferryboat; and the "locomotive" in the lightning trains; and these, he tells us, are correlatitudinarianebulated to crustyshallophilapidosophy, cellerylophistichophagy, and corporaloscholastifumigasophy, as the defunctorifferationary expressions of organic planetary forces.

It is possible Blinks is right. He wears very long hair; and appears to speak "from the fount of inspiration which wells up within" him; but we find it hard to give up a favorite theory so long cherished. W.

We understand that arrangements have been made for a very thorough course of lectures on the Microscope, in this city, during the coming winter, by Dr. Geo. E. Jones. It will have especial reference to the practical working of the Microscope, and also in its application to medical science. This subject is one to which the Dr. has given a large share of attention, and one the presentation of which in his hands will be very interesting and instructive to all for whom it has any attractions. T.

